# Advancing predictive, preventive, and personalized medicine in eyelid diseases: a concerns-based and expandable screening system through structural dissection

**DOI:** 10.1007/s13167-025-00401-y

**Published:** 2025-03-05

**Authors:** Jing Cao, Kun You, Peifang Xu, Yiming Sun, Ji Shao, Yifan Zhou, Huimin Li, Lixia Lou, Qi Miao, Juan Ye

**Affiliations:** 1 Eye Center of the Second Affiliated Hospital, School of Medicine, Zhejiang University, No.88 Jiefang Road, Hangzhou, 310009 Zhejiang China; 2 Zhejiang Feitu Medical Imaging Co., Ltd, Hangzhou, 310000 Zhejiang China

**Keywords:** Predictive preventive personalized medicine (PPPM / 3PM), Eyetome, Ophthalmology, Blepharoptosis, Thyroid eye disease, Eyelid tumors, Secondary targeted prevention, Deep learning, Interpretability, Expandability, Eyelid disorders, Screening, Monitoring

## Abstract

**Background/aims:**

Early recognition of eyelid morphological abnormalities was crucial, as untreated conditions could lead to blinding complications. An eyelid screening system that could provide both anatomical and pathological information was essential for formulating personalized treatment strategies. This study aimed to develop a clinically concerns-based framework capable of identifying common eyelid diseases requiring further intervention by evaluating individual anatomical and pathological changes. This approach would enhance individualized and efficient prevention, while supporting targeted treatment strategies.

**Methods:**

The eyelid disorder screening system, Eyetome, was developed based on a morphological atlas and comprised four modules designed to identify 14 common eyelid disorders and pathological changes. A total of 6180 eye patches were analyzed to extract anatomical and pathological features. The performance of Eyetome was evaluated using average accuracy (aACC) and F1 score, with comparisons made against traditional models and ophthalmologists. To assess the system’s expandability, an additional test was conducted in a multimorbidity scenario.

**Results:**

Eyetome demonstrated high performance in recognizing single diseases, achieving an aACC of 98.83% and an F1 score of 0.93. The system outperformed classic models, with an aACC of 98.83% compared to 96.72% for Desnet101 and 97.59% for Vit. Additionally, Eyetome’s aACC exceeded that of a junior ophthalmologist (JO) (97.11%) and was comparable to a senior ophthalmologist (SO) (98.69%). In the extended multimorbidity dataset, Eyetome maintained robust performance with an accuracy of 97.97%, surpassing JO (95.47%) and closely matching SO (97.81%).

**Conclusions:**

This study developed a clinical concerns-based system for screening and monitoring eyelid disorders, aimed at supporting predictive diagnosis, preventing diseases progression, and facilitating more effective, patient-centered treatment of common eyelid disorders, aligning with the principles of predictive, preventive, and personalized medicine (PPPM/3PM). The system’s interpretability, scalability, and user-friendly data acquisition design could further enhance its acceptance among both doctors and patients, facilitating the shift from reactive medicine to proactive precision medicine.

**Supplementary Information:**

The online version contains supplementary material available at 10.1007/s13167-025-00401-y.

## Introduction

Normal visual function and facial appearance depend heavily on the proper morphology and positioning of the eyelid. As the eyelid is anatomically close to the corneal surface, abnormalities can profoundly affect the ocular surface, resulting in conditions such as exposure keratitis vision loss, and even cancer metastasis [1]. If left undiagnosed or untreated, these conditions can lead to complications such as amblyopia, corneal irritation, recurrent infections, and in severe cases, life-threatening outcomes. Moreover, they can cause significant physical and psychological distress, greatly diminishing a patient’s quality of life [2].

Eyelid diseases encompass a broad spectrum of conditions, including morphological abnormalities, inflammatory disorders, and malignancies. There are over 10 types of eyelid diseases [3] affecting over 13% of the global population [4], with only one specialist per 450,260 individuals even in developed countries [5]. It is difficult to precisely diagnose every patient, particularly in remote areas. State-of-the-art artificial intelligence (AI) has been employed in several studies to semi-automatically or automatically measure eyelid morphological parameters from photographs to identity eyelid disorders [6–8]. Additionally, some teams have developed deep learning (DL)-based models to automatically detected blepharoptosis [9], thyroid eye disease [10], and eyelid tumors [11]. However, a comprehensive screening system that can be applied to different types of eyelid disorders is still lacking.

Previous screening systems for multiple diseases were primarily constructed based on image-level labels [12, 13]. However, models that solely utilized image-level images as inputs could not consider all diseases from a unified perspective. These models might struggle to extend to new types of diseases and could fail when encountering classes that were not included in the training set. The lack of a transparency that could integrate AI insights into practice has significantly limited their clinical application within the framework of predictive, preventive, and personalized medicine (PPPM/3PM) [14]. This limitation hindered doctors from accessing personalized information provided by the model, thereby impeding their ability to make tailored, patient-specific decisions. While this challenge might be mitigated by adding images of target classes [15–17], it remained difficult to build a model encompassing almost all diseases and was applicable to patients with multimorbidity. Furthermore, the interpretation of models trained with image-level labels was limited. Heatmaps were proposed to visualize the attention of DL models [11, 18, 19], but it remained unclear whether the highlighted area represented a novel clinical finding or erroneous relevance [20, 21]. Thus, a unified screening system with improved expansibility and interpretability was essential for an automatic diagnosis in complex clinical settings.

### Working hypothesis and purpose in the framework of PPPM

There were critical gaps in the clinical diagnosis and management of eyelid disorders. Many eyelid abnormalities, including morphological changes and pathological conditions, often go unnoticed until they reach advanced stages, leading to delayed interventions and increased risks of complications. Furthermore, the complexity of diagnosing multiple coexisting eyelid disorders, particularly in patients with multimorbidity, underscores the need for a comprehensive, accurate, and interpretable screening tool. Realizing early detection, multimorbidity diagnosis, and disease progression monitoring of multiple eyelid disorders through advanced deep learning techniques was a critical step in preventing disease progression and complications, as emphasized by the European Association for PPPM/3PM in its white paper [22]. An anatomical-pathological visualization framework would enable clinicians to implement more personalized management strategies, yet such an approach remains underutilized in clinical practice. Therefore, an AI-based PPPM approach capable of accurately screening eyelid abnormalities and providing fine-grained, interpretable outputs was urgently needed in the clinical setting.

In this study, we proposed an eyelid disease screening system capable of recognizing 14 common eyelid morphological changes and diseases with an expansible and transparent framework based on a morphological atlas, to facilitate early detection and interventions, precise multimorbidity diagnosis, dynamic monitoring of disease progression, and prevent progressing from reversible conditions to irreversible severe pathologies and cascading collateral complications. By integrating fine-grained analysis and personalized outputs, Eyetome empowered clinicians to make more informed decisions, enhancing patient outcomes while aligning with the principles of predictive, preventive, and personalized medicine (PPPM/3PM). This innovative approach bridged existing gaps in clinical practice, particularly in settings with limited access to specialized ophthalmologists, offering a robust solution to improve diagnostic accuracy and patient care.

## Materials and methods

### Dataset acquisition and annotation

This retrospective study was conducted at the Second Affiliated Hospital of Zhejiang University, School of Medicine (SAHZU), and 4484 Asian patients were recruited between August 1, 2016, and July 31, 2022, including 3106 patients with eyelid diseases and 1378 patients with normal eyelid morphology. The study protocol was approved by the Human Research Ethics Committee (HREC) of SAHZU (No.2023–1279). All the procedures adhered to the principles of the Declaration of Helsinki. Informed consent was obtained from each patient, and written consent was exempted by the HREC of SAHZU because it was a retrospective study.

Eight common eyelid morphological changes [23, 24], including eyelid entropion and trichiasis, lower eyelid retraction, upper eyelid retraction, lower eyelid ectropion, tumor, ptosis, epicanthus inversus, and other types of epicanthus, were identified in this study. Six common eyelid diseases, including blepharoptosis, thyroid-associated ophthalmopathy (TAO), ectropion, eyelid tumor, entropion, trichiasis, blepharophimosis/ptosis/epicanthus inversus syndrome (BPES), and numerous multimorbidities, were further identified. Detailed definitions of the eyelid diseases were provided in the supplementary file. Patients with diagnoses of target diseases were included, and the exclusion criteria were as follows: (1) a history of corneal surgery or injury that would affect the normal appearance of the cornea, (2) equivocal diagnoses, and (3) poor compliance.

All photographs were taken using a digital single-lens reflex camera (Canon EOS 500D with a 100 mm macro lens, Canon Corporation, Japan) as a standard full-face photo. All participants were asked to gaze at the primary position with a circular marker 10 mm in diameter attached to the forehead as a standard reference for spatial distance. Only one photograph from each patient was selected, and all photographs were saved as JPG files with a resolution of 4752 by 3618.

An ophthalmologist with more than 6 years of clinical experience was invited to label the photos based on the types of pathological changes and anatomical regions. The ground truth for each eye was based on electronic medical records from ophthalmologists with more than 20 years of clinical experience.

### Development of the eyelid disorder screening system—Eyetome

**Fig. 1:**
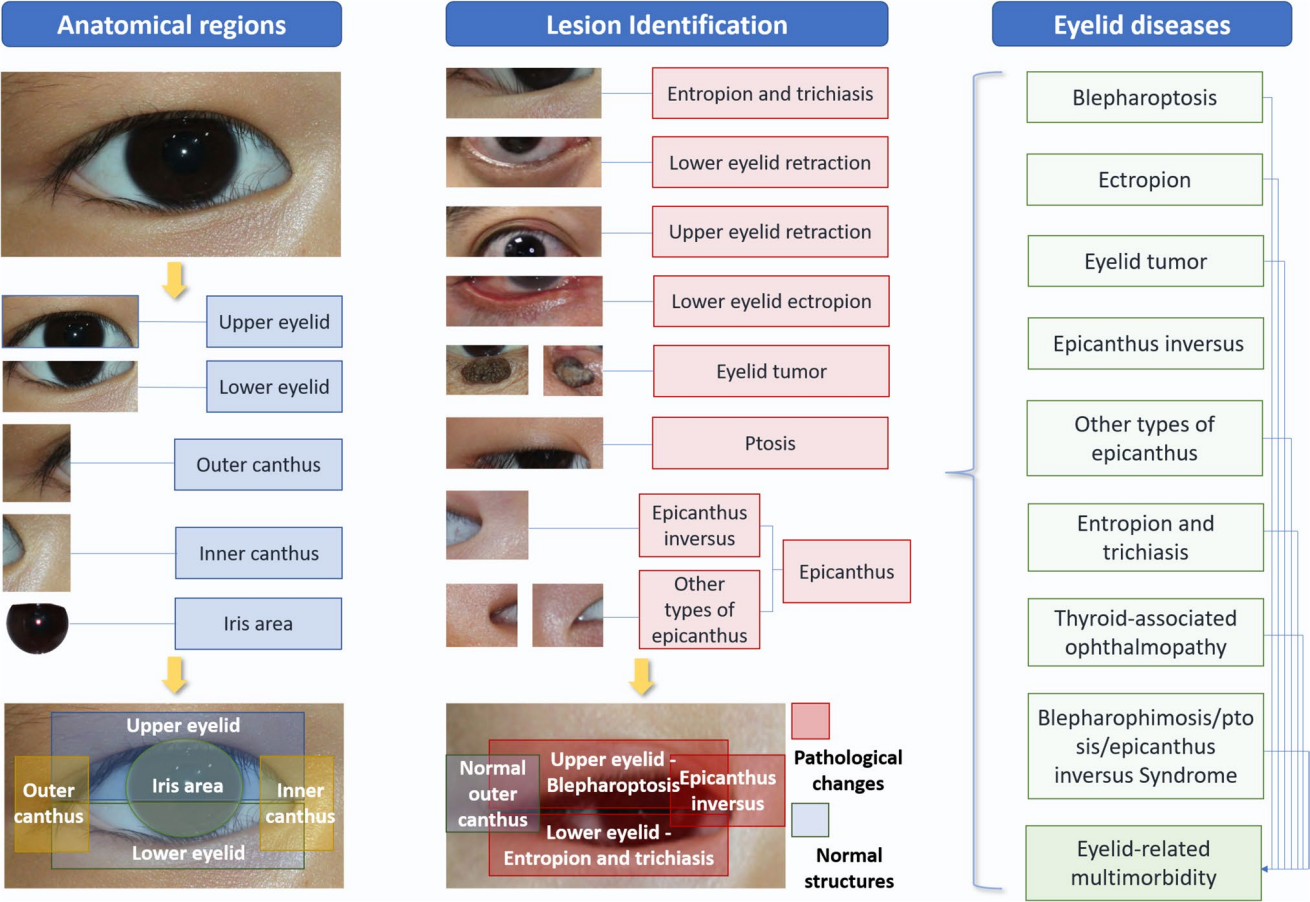
The architecture of the eyelid morphological atlas, which served as the foundational principle of Eyetome. The atlas was constructed based on the logical relationship between pathological changes and anatomical regions, enabling the precise diagnosis of eyelid diseases. Utilizing the morphological atlas, Eyetome firstly identified the anatomical regions and then discerned the pathological changes in each region to arrive at a final diagnosis

**Fig. 2:**
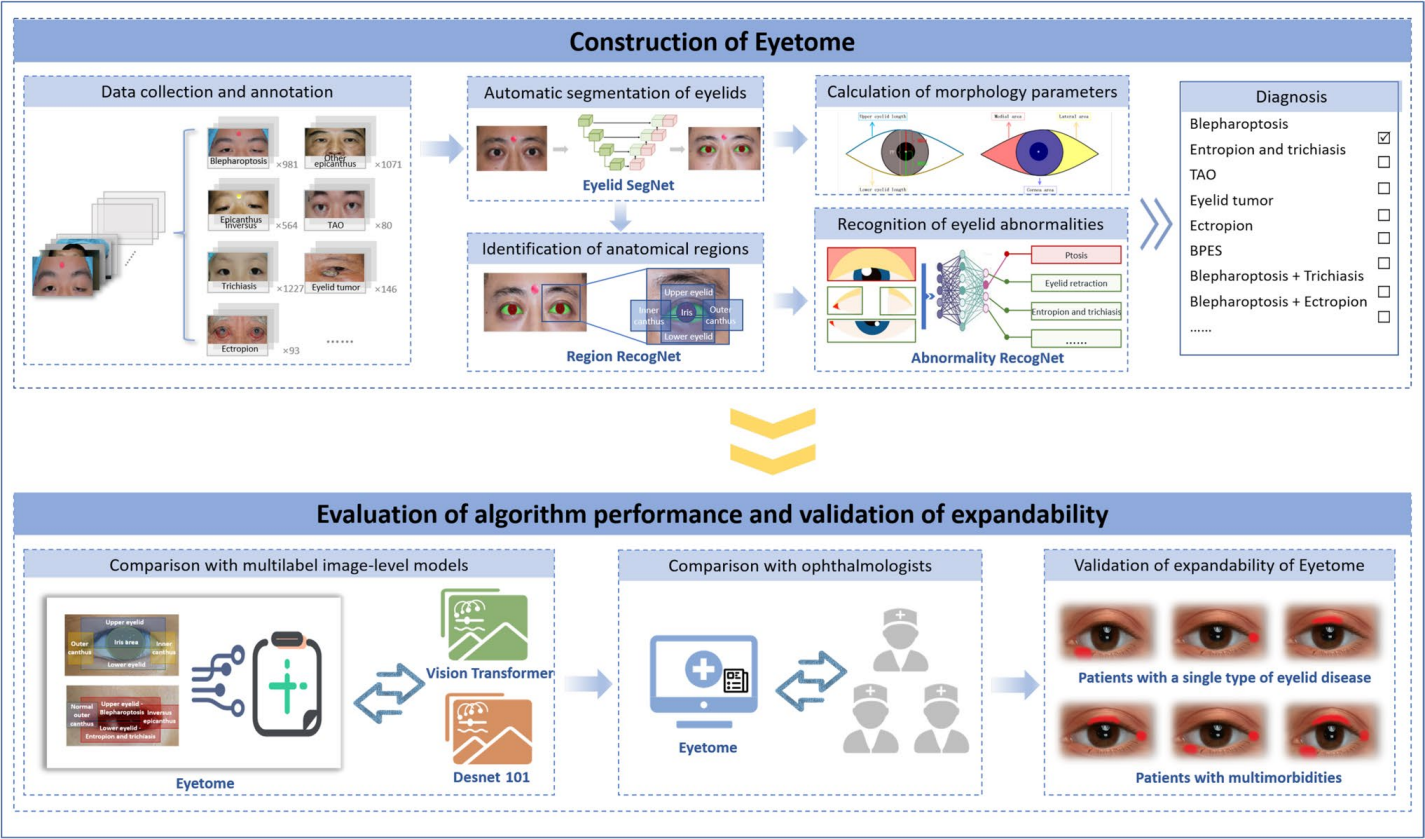
The main pipeline of the study. Eyetome was developed using the eyelid morphological atlas, which integrated pathological changes and anatomical regions within a clinically logical framework. Comparative tests with image-level models and human specialists were conducted to evaluate Eyetome’s stability and precision. An expanded test in multimorbidity scenarios was performed to further examine the system’s scalability and adaptability

Based on the clinical relationship between the pathological changes of the eyelid and their anatomical position, we designed a morphological atlas, which referred to the abstract architecture that could logically associate the pathological changes and regions, as the major principle of the Eyetome. It mimicked the way human ophthalmologists thought during clinical diagnosis to generate a diagnosis of a single disease and multimorbidity more intuitively and convincingly (Fig. 1). Eyetome consisted of the following four modules based on the morphological atlas: (1) an automatic eyelid segmentation module, (2) an anatomical region identification module, (3) a morphology parameter calculation module, and (4) an eyelid abnormality recognition module (Fig. 2).

The face recognition library [25] in Python 3.9 was applied to detect eyes in images. Monocular patches were generated according to the detection bounding boxes. The DL-based method we proposed previously [26] was used to construct the automatic eyelid segmentation module (Eyelid SegNet, the first module) for automatic segmentation of eyelids and the cornea area.

The anatomical region identification module (Region RecogNet, the second module) was developed by dividing the monocular patch into four anatomical regions according to the output of the Eyelid SegNet, including inner canthus region, outer canthus region, upper eyelid region, and lower eyelid region. The inner/outer canthus was defined as the pixel point on the contour line nearest/furthest from the midline of the nose. The canthus patch was defined as a canthus-centered square area with a length one-third of the distance between the inner and outer canthus. The upper/lower eyelid patch was the minimum bounding rectangle of the upper/lower eyelid contours.

The morphology parameter calculation module (the third module) was built for eight morphological parameter measurements as we described previously [26], including margin reflex distance (MRD) 1, MRD 2, palpebral fissure (PF), corneal area, lateral area, medial area, upper eyelid length, and lower eyelid length. Changes in eyelid parameters were used to screen for diseases, such as TAO and blepharoptosis, by setting parameter thresholds. This module could also be utilized to evaluate and monitor morphological changes throughout disease progression.

The eyelid abnormalities recognition module (Abnormality RecogNet, the fourth module), which took anatomical region patches as input, was aggregated by the inner canthus classifier, eyelid classifier, and tumor segmentator. The inner canthus classifier and eyelid classifier were based on the vision transformer (ViT) [27], which was a transformer architecture distinguished by its adoption of multi-head attention and position-wise feed-forward structure. It cut the input image into sequential embedded parts and differentiated the significance of each part. Furthermore, Attention U-Net was used as the backbone of the tumor segmentator. The attention gate (AG) mechanism in the model highlighted only the relevant activations and suppressed activations in irrelevant regions to reduce wasted computational resources. It focused more on the regions with higher weights and significantly improved the accuracy of tumor segmentation.

Finally, based on the rules of the morphological atlas, the four modules were aggregated into Eyetome, which identified eyelid diseases by relating the anatomical regions and different types of abnormalities. Considering that the diagnostic approach for eyelid diseases varied from parameter measurement to abnormal pattern recognition, Eyetome was designed in a concerns-based way against different diseases, which meant it could perform eyelid parameter calculation and abnormal pattern recognition at the same time, and could focus on specific abnormalities rather than show potential highlighted regions as traditional image-level models. Since Eyetome recognized diseases based on the abnormalities in each anatomical region, it could not only be applied to single diseases, but also to multimorbidity in a human-mimicking logic.

### Comparison with image-level identification models

To investigate the practicability of the morphological atlas, the performance of Eyetome was further compared to traditional multilabel models which used image-level data as input, including a multilabel Desnet101 [28] and a novel vision transformer (Vit) [27]. The models were trained, validated, tested, and fine-tuned on the same dataset as Eyetome.

### Extended validation in a multimorbidity scenario

Eyetome was supposed to precisely identify not only single diseases but also multimorbidity, which Eyetome has never been trained to screen before. Thus, extended validation of Eyetome was performed using an extra multimorbidity test set containing 640 multimorbidity photographs. The categories of multimorbidity photographs were not included in the training set, which meant that this was the first time Eyetome encountered such extended categories.

### Comparison of Eyetome with ophthalmologists

One junior ophthalmologist (JO) with more than 3 years of clinical experience and one senior ophthalmologist (SO) with more than 10 years of clinical experience outside the annotation team were invited to further evaluate the capability of Eyetome on the single disease test set and multimorbidity test set. They were asked to independently provide a diagnosis for each eye based on photographs. The results were then compared with the predefined gold standards.

### Statistical analysis

The receiver operating characteristic (ROC) curve and the area under the ROC curve (AUROC) were generated to evaluate the identification ability of eyelid morphological changes. Sensitivity, specificity, accuracy (ACC), F1 scores, and AUROC were introduced to quantify the performance of Eyetome, multilabel Desnet101, multilabel Vit, and human specialists. The parameters were calculated with the eye as the unit of measurement. A Chi-Squared test was applied to assess the performance of Eyetome and the clinicians in the extended test. Statistical significance was set at *p* < 0.05. All statistical analyses were conducted using GraphPad Prism 8.3.0 and Python 3.7.

## Results

### Data characteristics

A total of 6180 eyes from 4484 patients were included in this study, comprising 1378 normal eyes (one from each individual) and 4802 abnormal eyes. Among all the data, 1378 normal eyes and 4162 eyes with a single disease were randomly divided into training, validation, and test sets in a ratio of 3:1:1, and 640 eyes with multimorbidity were incorporated in the extended validation set to evaluate expandability. Demographic information and distribution of the dataset are shown in Table 1.

**Table 1:** Demographic statistics of the included participants

Total no. of images	4484			
No. of patients	4484			
No. of eyes (right:left)	6180 (2608:3572)			
Age, mean ± SD	47.50 ± 37.50			
No. of women (%)	2,438 (54.38)			
	Training set (eyes)	Validation set (eyes)	Test set (eyes)	Total (eyes)
Normal	828	275	275	1378
Blepharoptosis	589	196	196	981
Entropion and trichiasis	737	245	245	1227
Thyroid-associated ophthalmopathy	48	16	16	80
Eyelid tumor	88	29	29	146
Ectropion	55	19	19	93
Epicanthus				
Epicanthus inversus	338	113	113	564
Other types of epicanthus	643	214	214	1071
Blepharoptosis + trichiasis	NA	NA	178	178
Blepharophimosis/ptosis/epicanthus inversus Syndrome	NA	NA	30	30
Blepharoptosis + other types of epicanthus	NA	NA	428	428
Blepharoptosis + ectropion	NA	NA	4	4
Total	3326	1771	1747	6180

*NA* not applicable

### Performance of Eyetome in identifying a single disease

**Fig. 3:**
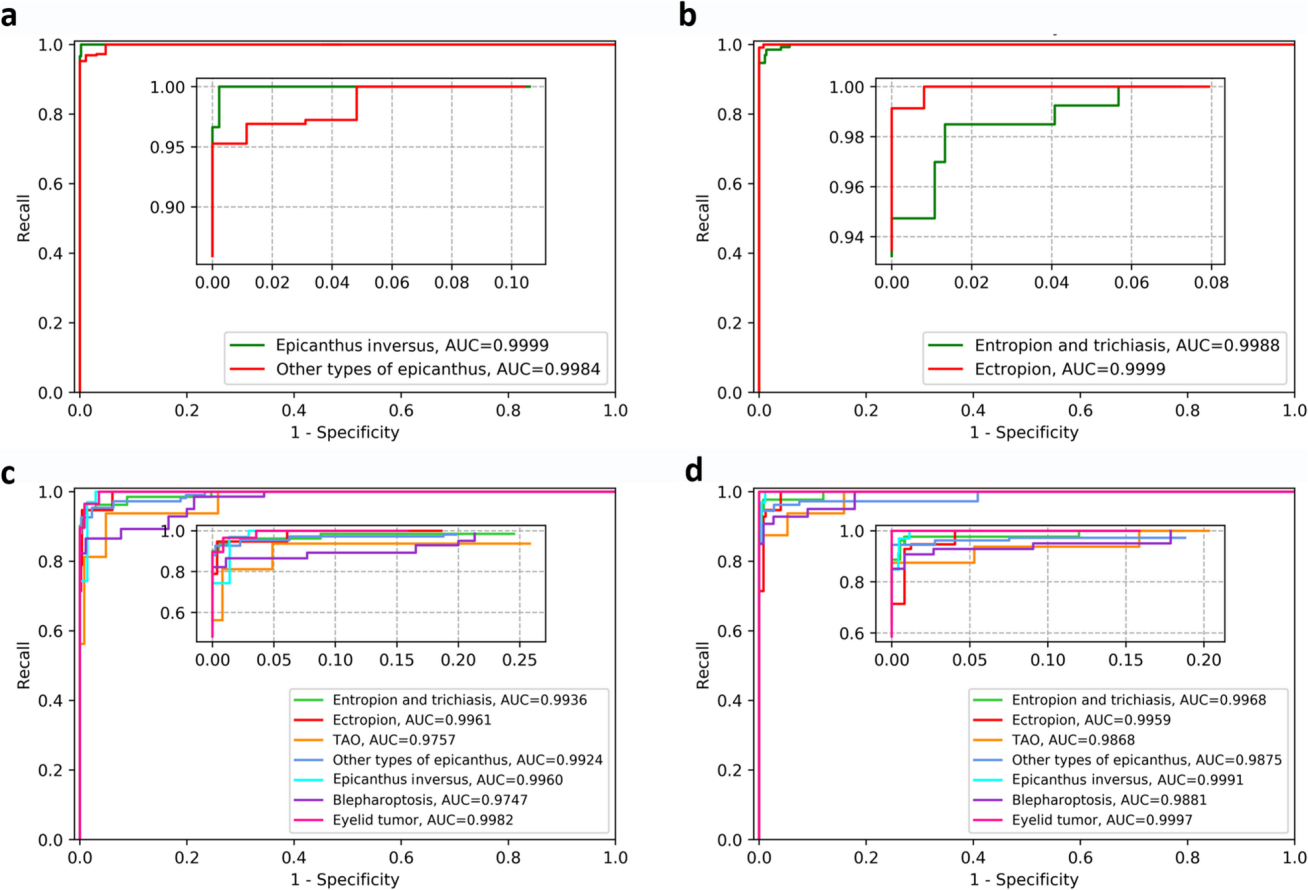
Receiver operating characteristic (ROC) curves of the Eyetome classifiers, including the inner canthus classifier (**a**) and the eyelid classifier (**b**), alongside those of image-level multilabel models, including Desnet101 (**c**) and ViT (**d**)

**Fig. 4:**
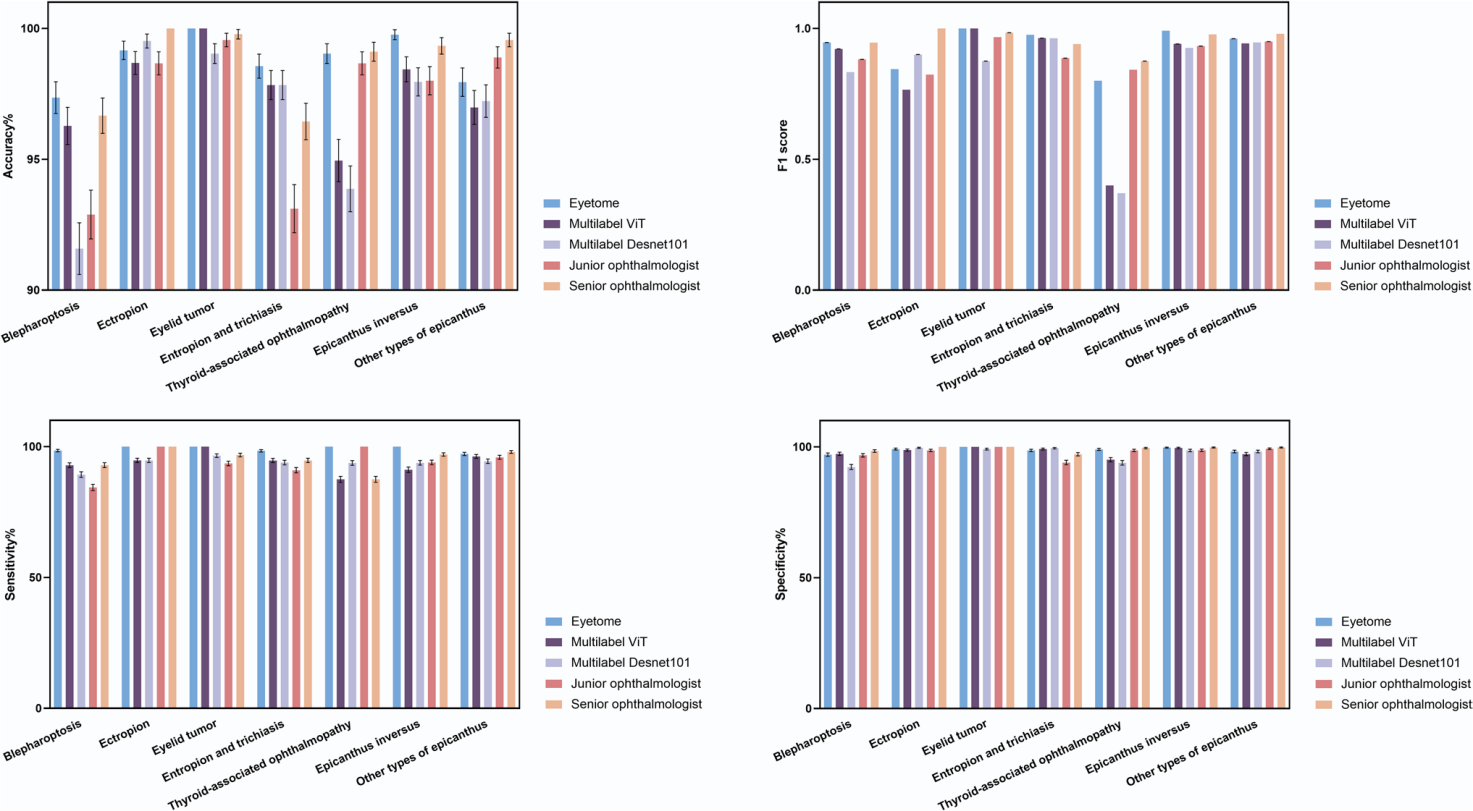
Comparison of accuracy, sensitivity, specificity, and F1 score between Eyetome, multilabel ViT, multilabel Desnet101, a junior ophthalmologist, and a senior ophthalmologist in identifying common eyelid diseases

The AUC for identifying eyelid morphological abnormalities was 0.9988 (95% CI 0.9975–1.0000) for entropion and trichiasis, 0.9999 (95% CI 0.9995–1.0000) for ectropion, 0.9999 (95% CI 0.9995–1.0000) for epicanthus inversus, and 0.9984 (95% CI 0.9968–1.0000) for other types of epicanthus (Fig. 3a and b). The ability of the abnormality recognition module to identify eyelid morphology abnormalities such as ptosis, eyelid retraction, and tumor was not evaluated independently because these abnormalities were classified based on parameter calculation and lesion segmentation. By integrating the segmentation module, calculation module, anatomical region identification module, and abnormalities recognition module, Eyetome reached an average ACC of 98.83% (95% CI 98.46–99.20%), sensitivity of 99.15% (95% CI 98.92–99.38%), specificity of 98.82% (95% CI 98.45–99.19%), and an F1 score of 0.93 (95% CI 0.92–0.94). All classes reached ACCs of over 97.3% and Eyetome performed best when recognizing eyelid tumors, with an ACC of 100.00%, followed by TAO (ACC = 99.04%, 95% CI 98.66–99.42%), ectropion (ACC = 98.56%, 95% CI 98.09–99.02%), entropion and trichiasis (ACC = 99.16%, 95% CI 98.80–99.51%), and blepharoptosis (ACC = 97.36%, 95% CI 96.73–97.98%). The highest sensitivity was reached for ectropion, TAO, and eyelid tumors (100.00%, 95% CI 100.00–100.00%). The best specificity was obtained for eyelid tumors, which was 100.00% (95% CI 100.00–100.00%) (Fig. 4, Table 2).

**Table 2:** The performance of Eyetome and comparison with classic algorithms

	Eyetome				Multilabel ViT				Multilabel Desnet101			
	ACC (%) (95% CI)	F1 (95% CI)	Sensitivity (%) (95% CI)	Specificity (%) (95% CI)	ACC (%) (95% CI)	F1 (95% CI)	Sensitivity (%) (95% CI)	Specificity (%) (95% CI)	ACC (%) (95% CI)	F1 (95% CI)	Sensitivity (%) (95% CI)	Specificity (%) (95% CI)
Blepharoptosis	97.36 (96.73–97.98)	0.95 (0.94–0.95)	98.47 (97.99–98.95)	97.01 (96.35–97.68)	96.27 (95.54–97.01)	0.92 (0.91–0.92)	92.86 (93.86–91.85)	97.33 (96.70–97.96)	91.59 (90.51–92.67)	0.83 (0.82–0.85)	89.29 (88.08–90.49)	92.30 (91.26–93.33)
TAO	99.04 (98.66–99.42)	0.80 (0.78–0.82)	100.00 (100–100)	99.02 (98.64–99.4)	94.95 (94.10–95.80)	0.40 (0.38–0.40)	87.50 (88.79–86.21)	95.10 (94.26–95.94)	93.87 (92.94–94.80)	0.37 (0.35–0.39)	93.75 (92.81–94.69)	93.87 (92.94–94.81)
Entropion and trichiasis	98.56 (98.09–99.02)	0.98 (0.97–0.98)	98.37 (97.87–98.86)	98.64 (98.19–99.09)	97.84 (97.27–98.40)	0.96 (0.96–0.96)	94.69 (95.57–93.82)	99.15 (98.79–99.51)	97.84 (97.27–98.40)	0.96 (0.95–0.97)	93.88 (92.94–94.81)	99.49 (99.21–99.77)
Ectropion	99.16 (98.80–99.51)	0.84 (0.83–0.86)	100.00 (100–100)	99.14 (98.78–99.5)	98.68 (98.23–99.12)	0.77 (0.75–0.77)	94.74 (95.61–93.87)	98.77 (98.34–99.2)	99.52 (99.25–99.79)	0.90 (0.89–0.91)	94.74 (93.87–95.61)	99.63 (99.39–99.87)
Eyelid tumor	100.00 (100–100)	1.00 (1.00–1.00)	100.00 (100–100)	100.00 (100–100)	100.00 (100–100)	1.00 (1–1)	100.00 (100–100)	100.00 (100–100)	99.04 (98.66–99.42)	0.88 (0.86–0.89)	96.55 (95.84–97.26)	99.13 (98.77–99.49)
Epicanthus inversus	99.76 (99.57–99.95)	0.99 (0.99–0.99)	100.00 (100–100)	99.72 (99.52–99.93)	98.44 (97.95–98.92)	0.94 (0.93–0.94)	91.15 (92.26–90.04)	99.58 (99.33–99.83)	97.96 (97.41–98.51)	0.93 (0.92–0.94)	93.81 (92.87–94.74)	98.61 (98.15–99.07)
Other types of epicanthus	97.95 (97.39–98.50)	0.96 (0.95–0.97)	97.20 (96.55–97.84)	98.21 (97.69–98.72)	96.98 (96.31–97.65)	0.94 (0.93–0.94)	96.26 (97–95.52)	97.23 (96.59–97.87)	97.22 (96.58–97.86)	0.95 (0.94–0.95)	94.39 (93.50–95.29)	98.21 (97.69–98.72)

### Comparison with image-level identification models in identifying a single disease

The effectiveness of the morphological atlas was further proven in the comparison test between Eyetome and other two image-level models that were trained on data without extracting anatomical-level information about morphological changes. It was shown that Eyetome exhibited better performance than multilabel Desnet101 and multilabel Vit, with an average ACC of 98.83% (95% CI 98.46–99.20%) versus 96.72% (Desnet101, 95% CI 96.02–97.41%) and 97.59% (Vit, 95% CI 97.00–98.19%), respectively. More specifically, Eyetome (99.15%, 95% CI 98.92–99.38% for sensitivity and 98.82%, 95% CI 98.45–99.19% for specificity) achieved higher sensitivity and specificity than Desnet101 (93.77%, 95% CI 92.83–94.71% for sensitivity and 97.32%, 95% CI 96.69–97.95% for specificity) and Vit (93.89%, 95% CI 92.95–94.82% for sensitivity and 98.17%, 95% CI 97.64–98.69% for specificity) (Fig. 4, Table 2). The ROC curves for Desnet101 and Vit are shown in Fig. 3c and d, respectively.

### Comparison with human ophthalmologists in identifying a single disease

We also compared the performance of Eyetome with that of human specialists to verify its single-disease screening ability. Eyetome showed higher average ACC (98.83%, 95% CI 98.46–99.20% versus 97.11%, 95% CI 96.46–97.76%), sensitivity (99.15%, 95% CI 98.92–99.38% versus 94.11%, 95% CI 93.19–95.03%), and specificity (98.82%, 95% CI 98.45–99.19% versus 97.99%, 95% CI 97.45–98.54%) than that of JO. The capability of Eyetome was close to that of SO, with an average ACC of 98.83%, 95% CI 98.46–99.20% (Eyetome) versus 98.69%, 95% CI 98.26–99.14% (SO) and an average specificity of 98.82%, 95% CI 98.45–99.19% (Eyetome) versus 99.22%, 95% CI 98.88–99.57% (SO). It showed that Eyetome was more sensitive to identifying abnormalities such as blepharoptosis, eyelid tumor, entropion and trichiasis, TAO, and epicanthus inversus identification than JO and SO (Fig. 4, Table 2).

### Expandability of Eyetome in a multimorbidity scenario

**Fig. 5:**
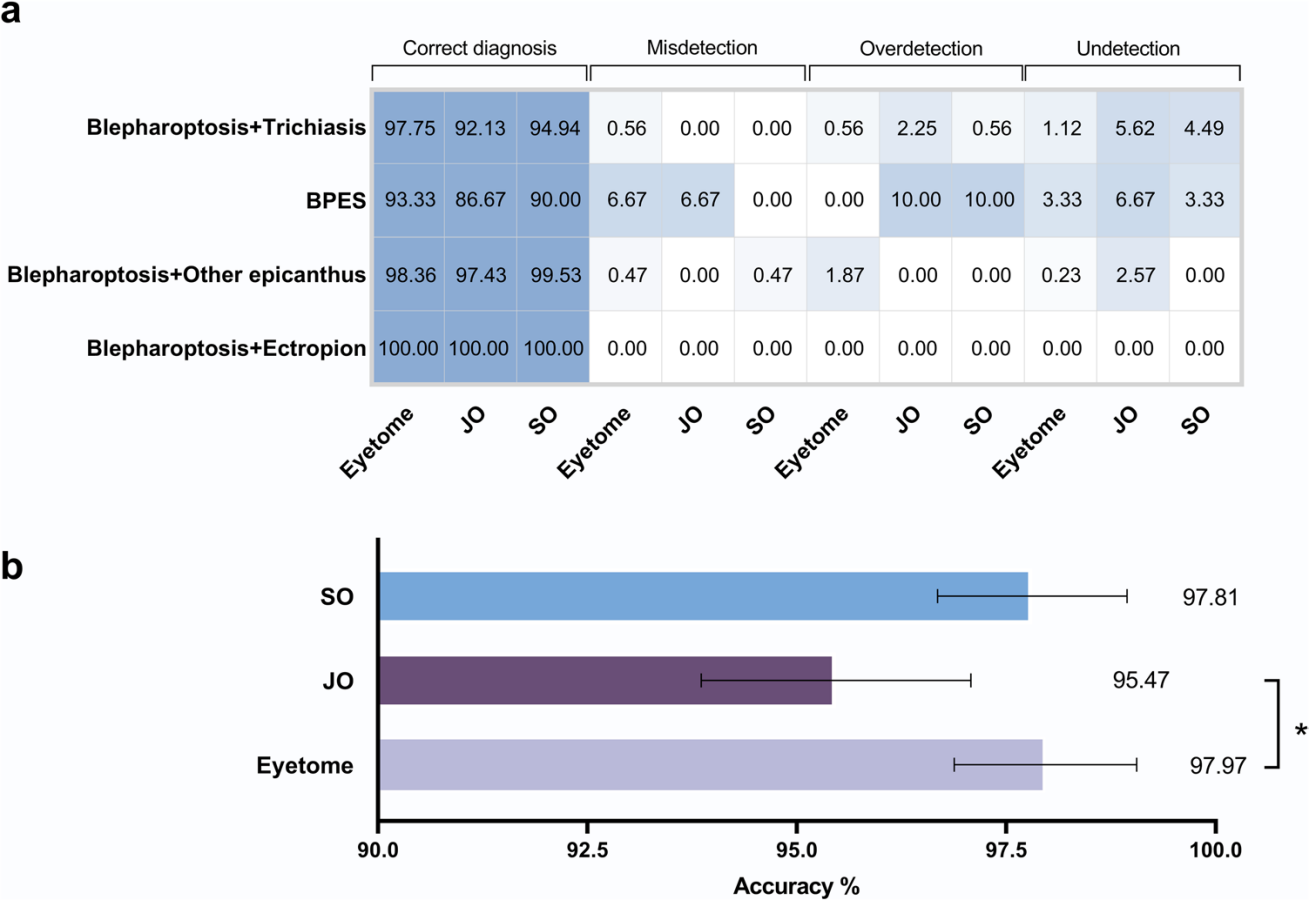
Performance comparison of Eyetome, junior ophthalmologist (JO), and senior ophthalmologist (SO) in diagnosing multimorbidity. The log10 fold-change heatmap (**a**) illustrates the distribution of correct diagnoses, misdetections, over-detections, and under-detections by Eyetome, JO, and SO across four multimorbidity classes. Log10 fold-change was calculated based on the proportion of correct diagnoses, misdetections, over-detections, and under-detections in each class, as indicated in the heatmap. Eyetome demonstrated higher accuracy in identifying multimorbidity compared to JO and achieved performance comparable to SO (**b**). *p < 0.05. BPES, blepharophimosis/ptosis/epicanthus inversus syndrome

Furthermore, we investigated whether the system was capable of recognizing a new phenotype in the image that the system had never encountered before. Eyetome was tested with 640 eyes that had more than one type of disease and exhibited a solid performance with an ACC of 97.97% (95% CI 96.88–99.06%). The ACC of Eyetome was significantly higher than that of JO (95.47%, 95% CI 93.86–97.08%, *p* = 0.018) and comparable to that of SO (97.81%, 95% CI 96.68–98.95%, *p* = 0.500). The number of undetected cases in Eyetome decreased from 23 to 4 compared with JO and decreased from 9 to 4 compared with SO (Fig. 5). It was found that the cases JO and SO missed were mainly focused on eyes with blepharoptosis and trichiasis. The overdetection cases of Eyetome were greater than those of JO and SO, and they were found to mainly exist in eyes with blepharoptosis and other types of epicanthus.

## Discussion

A holistic assessment and treatment plan development of patients with eyelid diseases depended not only on the disease types but also on the abnormality’s location and degree. Thus, an explicable screening system that can provide detailed information and can be applied to both single diseases, and multimorbidity is more reasonable for predictive diagnosis and a more personalized management in the context of PPPM/3PM [29]. In this study, we innovatively mimicked the clinical logic deduction by analyzing eyelid anatomical and pathological changes and developed a screening system that could cover 14 common eyelid diseases and evaluate morphological changes during self-monitoring. The system achieved an average ACC of 98.71% in the single-disease diagnostic scenario and 97.97% in the expanded multimorbidity scenario. This study could present a valuable methodology for the early detection of eyelid diseases, helping to prevent potentially blinding complications and enabling personalized treatment strategies.

To accurately identify various eyelid diseases, facilitate the timely detection of conditions requiring intervention and assess disease progression, a model capable of precisely recognizing common clinical disease types and evaluating eyelid morphology is essential. While previous studies have focused on identifying specific eyelid diseases such as TAO [30] and ptosis [9, 31–33], no research to date has attempted to accurately identify multiple common eyelid diseases simultaneously. To adapt the system to accurately recognize different types of common eyelid diseases and evaluate the morphological changes, we assembled four modules to automatically calculate eyelid morphological parameters, recognize and locate abnormalities, and identify segment lesions. The integrated screening framework, Eyetome, was proven to be efficient in diagnosing all types of eyelid diseases, with ACC ranging from 97.36 to 100.00%, and F1 scores from 0.8 to 1.0 in diagnosing all types of eyelid diseases. It achieved the highest ACC in eyelid tumors (100%) owing to the lesion segmentation. Segmentation was individually designed for tumors because the size of the tumor should be considered when doctors made operative proposals. Eyetome also performed well in detecting diseases with morphological changes, with an average ACC from 97.95 to 99.76%. The lowest ACC was achieved in blepharoptosis (97.36%), which probably because a minor millimeter-level bias could lead to incorrect judgement of diseases diagnosed by eyelid parameter changes. However, the ACC of Eyetome in identifying blepharoptosis was still excellent compared to previous studies that achieved ACC values of 82.8% [30], 84.78% [9], and 90% [34]. Instead of image-level classification and extraction of eyelid contour key points for parameter measurement, as reported previously [9, 30, 34], we designed a DL-based methodology to assist eyelid segmentation and significantly improve the performance of Eyetome in classifying diseases diagnosed by parameter changes. The advantage was also proven in TAO, with an ACC of 86% in a previous image-level classification study [7] versus 99.04% in our study. Eyetome also showed better performance in a comparative test with multilabel image-level models (Fig. 3, Table 2), which confirmed that the specific morphological-change-oriented analysis framework could decrease irrelevant noise, amplify crucial features, and improve the ability to screen different forms of eyelid diseases accordingly. It held significant value for early detection within the framework of PPPM/3PM [35], helping to prevent eyelid diseases from progressing to severe complications, including blindness.

A clinically acceptable DL-based screening system should be transparent and interpretable to ophthalmologists, which meant that they could capture the concerns of models and comprehend the inference process. By visualizing and explaining the critical areas of the model’s focus, Eyetome could help clinicians identify early lesions or subclinical conditions, improving the sensitivity of disease prediction, thus facilitating the shift from reactive medicine to proactive precision medicine, aligning with PPPM/3PM [36]. The “black box” problem of existing deep learning models strongly restricted the clinical application of AI. Although methodologies have been developed to overcome this problem, such as occlusion testing [37] and class activation mappings (CAMs) [38], the highlighted area was sometimes not clinically relevant [39], and it was difficult to determine whether the area was a new biomarker or just an improper relevance [40]. To facilitate clinical understanding of predicted results from models, Eyetome simulated the clinicians’ reasoning to diagnose eyelid diseases by firstly locking the target abnormality, and then combining its location, degree, and type to output the final decision in an evidence-visualized way (Fig. 1). Thus, ophthalmologists could make a more reliable diagnosis by combining their experience and the pathological changes provided by Eyetome.

Another advantage of Eyetome was its extensibility to new, untrained phenotypes, such as multimorbidity, allowed it to adapt to diverse clinical scenarios, including rare or complex cases. This adaptability ensured early and comprehensive identification of conditions requiring intervention, reducing the risk of severe complications. By integrating new disease types based on clinical needs, Eyetome supported the proactive development of prevention strategies tailored to evolving patient populations. Previously, numerous studies have been conducted on the automatic diagnosis of eyelid diseases [11, 38, 41], but they were isolated. Thus, a morphological atlas was designed to separate the eyelid region into anatomical parts, recognize abnormalities in each part, and combine the anatomical and morphological information. Thus, all eyelid diseases could be regarded from a unified perspective, and Eyetome could theoretically integrate other types of diseases according to clinical requirements more easily. Consequently, the extensibility of Eyetome in new phenotypes that it has not been trained on before, such as in patients with multimorbidity, could be improved. Most of the models constructed in previous studies could classify only one kind of eyelid disease [42, 43] and were difficult to apply to patients with multimorbidity, which were common in the clinic. Considering that the identification of multimorbidity would be challenging for multilabel models because of the noise and intertwined characteristics, we developed a system that could essentially discriminate abnormalities in different diseases. It was proven that Eyetome, which contained lesion-level analysis modules, performed stably when applied to the extended multimorbidity scenario, with an ACC of 97.97%, which was comparable to that of SO.

## Conclusions and expert recommendations

We developed a clinical concerns-driven automatic screening system for eyelid diseases. This system integrated visualized anatomical and pathological features to enable precise treatment planning from a unified perspective, making it suitable for both single-disease and multimorbidity scenarios. The design of this interpretable screening system was closely aligned with the principles of PPPM/3PM. By providing accurate diagnostics and early warnings, the system advanced preventive healthcare. It supported personalized interventions through detailed assessments and dynamic monitoring while fostering effective communication between clinicians and patients via a transparent and expandable framework. Overall, this system established a robust foundation for delivering patient-centered healthcare, driving the practical implementation of PPPM/3PM and bridging the gap between theory and practice.

### Predictive medical approach

We integrated a morphological atlas into the system, enabling a unified analysis of all eyelid diseases. By visualizing and explaining the critical areas of the model’s focus, the screening system assisted clinicians in identifying early lesions or subclinical conditions. This capability supported the early detection of abnormalities and dynamic monitoring of disease progression, even in complex scenarios like multimorbidity, where multiple conditions coexist. Eyetome’s ability to address noise and overlapping features allowed it to distinguish abnormalities across various diseases effectively, significantly enhancing its predictive capabilities. This enabled clinicians to better anticipate complications and disease trajectories. Remarkably, Eyetome demonstrated superior performance compared to previous studies [7, 9, 30, 34], even when handling multiple tasks simultaneously. It achieved an average accuracy of 98.71% for common eyelid diseases and 97.97% for multimorbidity (Table 2). Moreover, by integrating individualized patient data with detailed evaluations of affected areas, the system could quantify and track disease dynamics. Therefore, this robust capability offered invaluable support for predicting disease trajectories, making Eyetome a powerful tool in predictive medicine.

### Targeted prevention

Eyetome demonstrated the ability to clearly identify early abnormalities and associated risks, enabling clinicians to devise targeted secondary prevention strategies and intervene before disease progression. By providing accurate disease risk assessments, the system supported healthcare providers in efficiently allocating diagnostic and therapeutic resources, thereby minimizing the risks of both overdiagnosis and missed diagnoses. This personalized approach not only facilitated timely interventions but also enabled the development of preventive measures tailored to each patient’s unique needs, significantly reducing the likelihood of complications, including vision impairment. With its precision and adaptability, the screening system has the potential to serve as a powerful tool for implementing predictive, preventive, and personalized medicine (PPPM/3PM) [44], ultimately improving patient outcomes while optimizing resource utilization in clinical practice.

### Personalized treatments

Eyetome was capable of recognizing up to 14 common eyelid morphological changes and diseases, offering refined diagnostic insights by analyzing the type, severity, and anatomical location of abnormalities. This detailed analysis ensured that the system remained transparent while enabling treatment plans to be closely tailored to each patient’s specific needs. Another key advantage of Eyetome’s transparency was its ability to seamlessly integrate AI-generated analyses with clinical expertise, enhancing the interpretability of diagnostic processes and fostering multidisciplinary collaboration. Furthermore, the system’s robustness in handling multimorbidity scenarios—commonly encountered in clinical practice—ensured that treatment decisions remained personalized even in complex cases. Eyetome’s personalized approach went beyond diagnosis, enabling targeted preventive measures and tailored follow-up strategies that addressed the unique needs of each patient. By combining precision, adaptability, and a patient-centered design, Eyetome provided a comprehensive framework for the personalization of medical services.

### How does the presented study contribute to the paradigm shift from reactive to PPPM/3P medicine and go beyond the state of the art?

This study advanced the paradigm shift from reactive medicine to PPPM/3PM by introducing Eyetome, a groundbreaking screening system that integrates a morphological atlas for unified analysis of eyelid diseases. Eyetome exceled in early detection, subclinical diagnosis, and dynamic morphological monitoring, enabling predictive insights into disease progression and complications, particularly in complex multimorbidity scenarios. Its transparent design fosterer clinician trust by visualizing critical areas and simulating clinical reasoning, while its personalized approach tailored treatment plans based on the type, severity, and location of abnormalities. With superior accuracy (98.71% in the single-disease and 97.97% in multimorbidity cases), Eyetome surpassed traditional models [9, 38, 41–43] by addressing multiple diseases simultaneously and providing actionable, patient-specific insights. By combining predictive diagnostics, preventive strategies, and personalized care, Eyetome transforms PPPM/3PM principles into practical applications, improving patient outcomes and optimizing healthcare resource allocation.

### Limitations and outlooks in the context of PPPM/3 M

This study has several limitations. Firstly, the dataset was imbalanced and more data should be collected in the future. Secondly, there was still a deviation in evaluating millimeter-level parameters, which could lead to the misdiagnosis of diseases such as TAO and blepharoptosis. Thirdly, the methodology needed to be improved to allow a more reliable calculation of eyelid parameters from photographs. Fourthly, all photographs were obtained from one camera, and the performance of Eyetome using external data from other types of equipment required further investigation.

Eyetome was the first of its kind to automatically recognize eyelid diseases in a quick-achieved manner. This aligned with the core principle of the PPPM/3PM approach, which emphasized quantifying and continuously monitoring patients’ clinical parameters to improve treatment outcomes [45]. To advance the future application of deep learning in this field, it was recommended to focus on the following key points:

1. Expanding the application of Eyetome from Asians to other populations to broaden its usability and impact.
2. Integrating detailed and targeted treatment strategies for each eyelid disorder with precise diagnosis and patient-specific risk assessments. It could enable the provision of comprehensive recommendations, spanning from diagnosis to treatment.

## Supplementary Information

Below is the link to the electronic supplementary material.

Supplementary file1 (DOCX 28 KB)

## Data Availability

Due to the privacy of patients, the data related to patients are not available for public access but can be obtained from the corresponding author on reasonable request approved by the Human Research Ethics Committee of the Second Affiliated Hospital of Zhejiang University.

## Abbreviations

AI: Artificial intelligence
AG: Attention gate
BPES: Blepharophimosis/ptosis/epicanthus inversus syndrome
DL: Deep learning
HREC: The Human Research Ethics Committee
MRD: Margin reflex distance
PF: Palpebral fissure
PPPM/3PM: Predictive, preventive, and personalized medicine
SAHZU: The Second Affiliated Hospital of Zhejiang University, School of Medicine
TAO: Thyroid-associated ophthalmopathy
