# Supplementary material for: Advancing predictive, preventive, and personalized medicine in eyelid diseases: a concerns-based and expandable screening system through structural dissection
Source: EPMA J. 2025 Mar 5;16(2):387–400. doi: 10.1007/s13167-025-00401-y (PMC12106165; doi:10.1007/s13167-025-00401-y)
Supplement: Supplementary file 1 — Supplementary file1 (DOCX 28 KB) [file 13167_2025_401_MOESM1_ESM.docx]

**Su**pplementary Information

**Definition and diagnosis criteria of the 6 included diseases and 8 morphological changes**.

1. **Pathological lesions**

**Eyelid entropion and trichiasis**

Eyelid entropion is an inward turning of the eyelid margin together with eyelashes growing in a posterior direction toward the corneal surface^[1]^.

**Lower eyelid retraction**

Lower eyelid retraction is a malposition of the lower eyelid, where the lid margin is displaced inferiorly. Typically, the lower eyelid rests at the level of the inferior corneal limbus, which is the border between the lower cornea and the sclera^[2]^. We define the patients with the lower eyelid beneath the inferior corneal limbus as lower eyelid retraction in this study.

**Upper eyelid retraction**

Upper eyelid retraction is a malposition of the upper eyelid, where the lid margin is displaced superiorly. Generally, the upper lid covers 1.0 mm of the superior part of the cornea. Since the normal average MRD1 is reported to be 4.5 mm, we define upper eyelid retraction as patients with MRD1 over 5 mm in this study^[3]^.

**Lower eyelid ectropion**

Lower eyelid ectropion is defined as the outward turning of the lower eyelid margin^[4]^.

**Eyelid tumor**

Eyelid tumor includes benign tumors and malignant tumors happening in either upper eyelid, lower eyelid, inner canthus or outer canthus, such as pigmented nevus, basal cell carcinoma, squamous cell carcinoma, etc^[5]^.

**Ptosis**

Normally, the upper lid covers 1.0 mm of the superior part of the cornea^[6,7]^ and the normal average age marginal reflex distance 1 (MRD1), which is the distance between the center of the pupillary light reflex and the upper eyelid margin with the eye in primary gaze, is reported to be 4.5 mm^[8]^. The patients with MRD1 less than 4.5 mm are defined as upper eyelid ptosis^[7,8]^.

**Epicanthus inversus**

Epicanthus inversus refers to a skin fold arising from the lower eyelid and ascending to the upper eyelid, which obscures the medial canthal structures^[9,10]^.

**Other types of epicanthus**

Epicanthal folds are oblique or vertical folds from the upper or lower eyelids towards the medial canthus. There are 4 types of epicanthus: Epicanthus inversus; Epicanthus tarsalis, referring to folds most prominent along upper eyelid; Epicanthus palpebralis, referring to folds involving both upper and lower eyelids; Epicanthus superciliaris, referring to fold originating from the brow and follows down to the lacrimal sac^[11]^. We define other types of epicanthus as epicanthus except epicanthus inversus in this study.

1. **Diseases**

**Blepharoptosis**

Blepharoptosis is an abnormal low-lying upper eyelid margin with the eye in primary gaze^[7]^. We define the patients with upper eyelid ptosis as blepharoptosis in this study.

**Thyroid-associated ophthalmopathy (TAO)**

TAO is an autoimmune disease caused by the activation of orbital fibroblasts by autoantibodies directed against thyroid receptors and the most common presenting sign of TAO is eyelid retraction^[12]^. In this study, we detect lower/upper eyelid retraction and define patients with lower/upper eyelid retraction as TAO patients.

**Ectropion**

Ectropion is an outward turning of the eyelid margin^[4]^.

**Eyelid tumor**

Eyelid tumor refers to patients with benign tumors and malignant tumors happening in either upper eyelid, lower eyelid, inner canthus or outer canthus ^[5]^.

**Entropion and trichiasis**

Entropion is an inward turning of the eyelid margin and appendages so that the pilosebaceous unit and mucocutaneous junction are directed posteriorly towards the cornea and ocular surface. Trichiasis is a condition in which eyelashes grow in a posterior direction toward the corneal surface. Entropion could lead to trichiasis with the change of eyelid margin’s location. It could happen in both upper eyelid and lower eyelid^[1]^.

**Blepharophimosis-ptosis-epicanthus inversus syndrome (BPES)**

BPES is an inherited eyelid syndrome presenting with telecanthus, epicanthus inversus, and ptosis. Patients with bilateral epicanthus inversus and ptosis are defined as BPES in this study^[10]^.

**Reference**

1. Weber, A.C., Chundury, R.V., and Perry, J.D. Entropion. <https://eyewiki.org/Entropion#cite_note-26>.

2. Barmettler, A., and Kodali, S. Lower Eyelid Retraction. <https://eyewiki.org/Lower_Eyelid_Retraction>.

3. Ben Simon, G.J., Mansury, A.M., Schwarcz, R.M., Lee, S., McCann, J.D., and Goldberg, R.A. (2005). Simultaneous orbital decompression and correction of upper eyelid retraction versus staged procedures in thyroid-related orbitopathy. Ophthalmology *112*, 923-932. 10.1016/j.ophtha.2004.12.028.

4. Belliveau, M.J. Ectropion. <https://eyewiki.org/Ectropion>.

5. Yen, M.T. Category:Oculoplastics/Orbit. <https://eyewiki.org/Category:Oculoplastics/Orbit>.

6. Bacharach, J., Lee, W.W., Harrison, A.R., and Freddo, T.F. (2021). A review of acquired blepharoptosis: prevalence, diagnosis, and current treatment options. Eye (London, England) *35*, 2468-2481. 10.1038/s41433-021-01547-5.

7. Alsuhaibani, A. Blepharoptosis. <https://eyewiki.org/Blepharoptosis>.

8. Putterman, A.M. (2012). Margin reflex distance (MRD) 1, 2, and 3. Ophthalmic Plast Reconstr Surg *28*, 308-311. 10.1097/IOP.0b013e3182523b7f.

9. Allen, C.E., and Rubin, P.A. (2008). Blepharophimosis-ptosis-epicanthus inversus syndrome (BPES): clinical manifestation and treatment. Int Ophthalmol Clin *48*, 15-23. 10.1097/IIO.0b013e3181694eee.

10. Alsuhaibani, A., and Onizan, T.A. Blepharophimosis Syndrome. <https://eyewiki.org/Blepharophimosis_Syndrome>.

11. Skurski, Z. Epicanthal Folds. <https://eyewiki.org/Epicanthal_Folds>.

12. FRCS, R.G., Durairaj, V.D., and Shah, K. Thyroid Eye Disease. <https://eyewiki.org/Thyroid_Eye_Disease>.
